# Immunogenicity and Efficacy of Flagellin-Fused Vaccine Candidates Targeting 2009 Pandemic H1N1 Influenza in Mice

**DOI:** 10.1371/journal.pone.0020928

**Published:** 2011-06-07

**Authors:** Ge Liu, Bart Tarbet, Langzhou Song, Lucia Reiserova, Bruce Weaver, Yan Chen, Hong Li, Fu Hou, Xiangyu Liu, Jason Parent, Scott Umlauf, Alan Shaw, Lynda Tussey

**Affiliations:** 1 VaxInnate Corporation, Cranbury, New Jersey, United States of America; 2 Institute for Antiviral Research, Utah State University, Logan, Utah, United States of America; Karolinska Institutet, Sweden

## Abstract

We have previously demonstrated that the globular head of the hemagglutinin (HA) antigen fused to flagellin of *Salmonella typhimurium fljB* (STF2, a TLR5 ligand) elicits protective immunity to H1N1 and H5N1 lethal influenza infections in mice (Song et al., 2008, PLoS ONE 3, e2257; Song et al., 2009, Vaccine 27, 5875–5888). These fusion proteins can be efficiently and economically manufactured in *E. coli* fermentation systems as next generation pandemic and seasonal influenza vaccines. Here we report immunogenicity and efficacy results of three vaccine candidates in which the HA globular head of A/California/07/2009 (H1N1) was fused to STF2 at the C-terminus (STF2.HA1), in replace of domain 3 (STF2R3.HA1), or in both positions (STF2R3.2xHA1). For all three vaccines, two subcutaneous immunizations of BALB/c mice with doses of either 0.3 or 3 µg elicit robust neutralizing (HAI) antibodies, that lead to > = 2 Log_10_ unit reduction in day 4 lung virus titer and full protection against a lethal A/California/04/2009 challenge. Vaccination with doses as low as 0.03 µg results in partial to full protection. Each candidate, particularly the STF2R3.HA1 and STF2R3.2xHA1 candidates, elicits robust neutralizing antibody responses that last for at least 8 months. The STF2R3.HA1 candidate, which was intermediately protective in the challenge models, is more immunogenic than the H1N1 components of two commercially available trivalent inactivated influenza vaccines (TIVs) in mice. Taken together, the results demonstrate that all three vaccine candidates are highly immunogenic and efficacious in mice, and that the STF2R3.2xHA1 format is the most effective candidate vaccine format.

## Introduction

Vaccination is a primary countermeasure to combat seasonal and pandemic influenza. Influenza vaccines have been manufactured as live attenuated, inactivated whole virus or split vaccines produced in embryonated hens' eggs using a method that was established over 60 years ago. Recently, mammalian cells have also been used to manufacture influenza vaccines in Europe. The major immunogens in these vaccines are viral hemagglutinin (HA) and neuraminidase (NA) proteins. While these vaccines are effective, they are inefficient to produce in terms of time and manufacturing capacity. When a pandemic influenza emerges for instance, it takes approximately 4–6 months from virus identification to production of the first vaccine doses. The collective experience with vaccine production for the last three influenza pandemics, particularly the 2009 H1N1 pandemic, has demonstrated that this timing is insufficient to meet global needs. Although dose sparing and increased longevity of immunity have recently been demonstrated when these vaccines are formulated with adjuvants (AS03 or MF59) [1], [2], a next generation technology for rapid production of pandemic influenza vaccines is still urgently needed [3], [4].

Recombinant technologies alleviate many of the production and capacity constraints associated with current technologies and provide a solution to global seasonal and pandemic influenza vaccine needs. These vaccine platforms include production of either recombinant HA [5] or virus-like particles consisting of HA, NA and matrix (M1) proteins [6], [7], vaccinia virus based expression of HA and NA [8], *E. coli* based expression of flagellin-HA globular head fusion proteins [9], the HA1 fragment of HA [10], or the HA2 stalk of HA [11], as well as DNA vector-based expression of multiple antigens [12].

VaxInnate's vaccine platform effectively links innate and adaptive immunity by genetically fusing the immunogen to flagellin of *Salmonella typhimurium (fljB)* (STF2). This flagellin fusion vaccine technology allows rapid development of vaccine seed clones in a couple of weeks, followed by economical manufacturing of the fusion protein-based vaccines using a well-established *E. coli* fermentation system and a standardized purification process. Flagellin is a TLR5 ligand consisting of domains 0, 1, 2, and 3, where domain 1 contains the TLR5-binding site [13]. We have demonstrated that the majority of conformational epitopes of the HA globular head of an H1N1 virus can be faithfully restored in a refolded fusion protein [14]. Simultaneous antigen delivery and TLR5 signaling to the same antigen presenting cells are believed to result in enhanced antigen presentation and induction of humoral and cell-mediated immune responses.

We have developed a panel of influenza vaccine formats which differ in the position of attachment of the HA globular heads fused to flagellin at different positions. Our initial vaccine formats fused the HA globular head of H1N1 subtypes to the carboxyl-terminus of flagellin. These seasonal vaccine candidates were highly immunogenic and protected mice against a lethal H1N1 influenza infection in mice [14]. The C-terminal format of the A/Solomon Islands/03/2006 HA globular head vaccine has also been shown to be well-tolerated and immunogenic in humans [15]. Subsequent development of pandemic H5N1 vaccines led to identification of a more efficacious alternative format of the vaccine, in which the HA globular head replaced the domain 3 of flagellin (STF2R3.HA5) [9]. Here we report promising preclinical immunogenicity and efficacy results of three vaccine candidates in which the HA globular head of A/California/07/2009 (H1N1) was fused to STF2 at the C-terminus (STF2.HA1), in replace of domain 3 (STF2R3.HA1), or in both positions (STF2R3.2xHA1).

## Results

**Figure 1:**
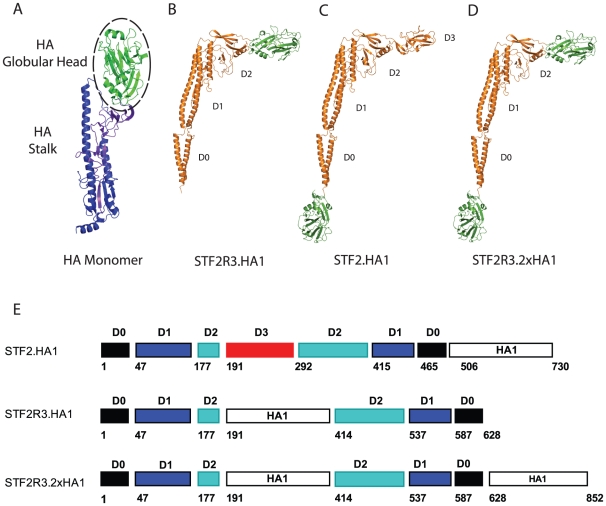
Ribbon diagram and schematic representation of the flagellin-HA globular head fusion vaccine candidates. HA globular head and flagellin structures are shown in green and orange, respectively. (**A**) HA monomer with encircled HA globular head; the HA globular head (HA1: amino acids 63–285) was genetically fused to the hypervariable region to replace domain 3 of flagellin (STF2R3.HA1, **B**), the C-terminus (STF2.HA1, **C**), or both positions (STF2R3.2xHA1, **D**). (**E**) linear schematic representation of recombinant flegallin-HA1 fusion proteins: Indicated are the flagellin domains D0 (black), D1 (blue), D2 (green), and D3 (red) as well as HA1 (open box) of Influenza A/California/07/2009. Beginning amino acid numbers of each domain are given.

With the emergence of the 2009 H1N1 pandemic we designed, produced and tested three different formats of the flagellin-HA vaccine candidates based on the A/California/07/2009 isolate (Fig. 1). A goal was to evaluate the timing to vaccine production, the efficiency of production and the relative immunogenicity and efficacy of the different vaccine formats in a preclinical model. The far left panel shows a ribbon diagram of the structure of influenza HA with the HA1-2 domain (HA1) highlighted in green (Fig. 1A). STF2R3.HA1 consists of one copy of HA in placement of the D3 domain of STF2 (STF2R3.HA1, Fig. 1B & 1E). STF2.HA1 consists of one copy of HA fused to the C terminus of STF2 (STF2.HA1, Fig. 1C & 1E). STF2R3.2xHA1 consists of one copy of HA fused to the C terminus of STF2 and the second in replacement of the D3 domain (STF2R3.HA1.2x, Fig. 1D & 1E).

**Figure 2:**
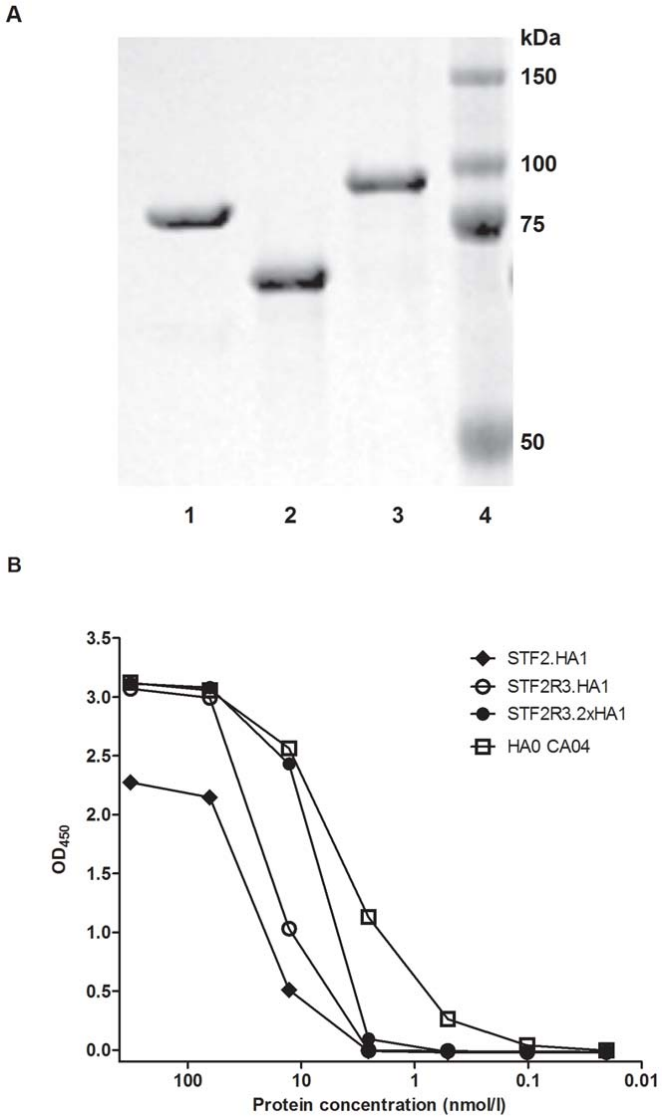
SDS-PAGE and antigenicity analyses of three purified recombinant. vaccine candidates. (**A**) Purified recombinant proteins were separated on an 4–12% SDS PAGE (0.5 µg protein/lane) and stained with Coomassie Blue. Lane1: STF2.HA1; lane 2: STF2R3.HA1; lane 3, STF2R3.2x HA1; and lane 4, Protein Marker. (**B**) Reactivity of ferret post infection serum to various vaccine candidates or reference antigen. ELISA plates were coated with serially diluted various CA07 proteins or HA CA04 (Protein Sciences) starting at 320 nmol/l in in PBS, reacted to ferret anti-CA7 serum, and detected with HRP-conjugated goat anti-ferret IgG. Mean OD_450_ of triplicates was read and graphed.

With regards to timing to vaccine production, we were able to progress from receipt of the HA gene sequence to purified proteins within 3 weeks. As shown in SDS-PAGE gel (Fig. 2A), purified STF2.HA1, STF2R3.HA1, and STF2R3.2xHA1 proteins were detected as single protein bands with predicted molecular weights of 77 kDa, 67 kDa, and 92 kDa, respectively (Fig. 2A). The purity as measured by reverse-phase HPLC and endotoxin level (measured by LAL test) of all lots used in this study are ≥95% and ≤5 EU/mg. All lots were ≥98% monomers by size exclusion chromatography, and had low levels of contaminating host nucleic acids and proteins. Finally, all three candidates induced significant dose-dependent IL-8 production in an *in vitro* TLR5 assay using HEK293 cells (Table 1).

**Table 1:** Purification step, yield, and flagellin bioactivity^a^ of candidate vaccines.

	STF2.HA1	STF2R3.HA1	STF2R3.2xHA1
**Expression**	75% soluble	Inclusion bodies	Inclusion bodies
**Refolding method**	Rapid dilution	Rapid dilution	Rapid dilution
**Chromatography**	AEX, SEC	AEX, SEC	AEX, SEC
**Yield (g purified protein/g cell past)**	1.43	1.79	1.67
**IL-8 induction (pg/ml)**	383±39	1002±164	553±35

a flagellin bioactivity was assessed by measuring secreted IL-8 with a sandwich ELISA (BD Biosciences) as previously described [9]. HEK293 cells in 96-well plates (n = 9) were stimulated with 278 ng/ml of each vaccine candidate for 16–18 hours at 37°C. IL-8 concentrations in medium were calculated from an IL-8 standard curve, and expressed as means ± SDs. IL-8 levels of cell control samples were < = 30 pg/ml.

To compare the antigenicity of the vaccine candidates, we examined the reactivity of the three fusion proteins with ferret post infection serum specific to A/California/07/2009 (CDC). All three candidates showed good specific antibody binding activities. As expected, the STF2R3.2x HA1 format showed the strongest reactivity after a full-length HA (HA0 CA04) expressed from baculovirus/insect cells to the ferret serum due to the presence of two copies of the globular head (Fig. 2B). The STF2.HA1 candidate displayed the weakest reactivity, and the STF2R3.HA1 format showed intermediate reactivity.

**Figure 3:**
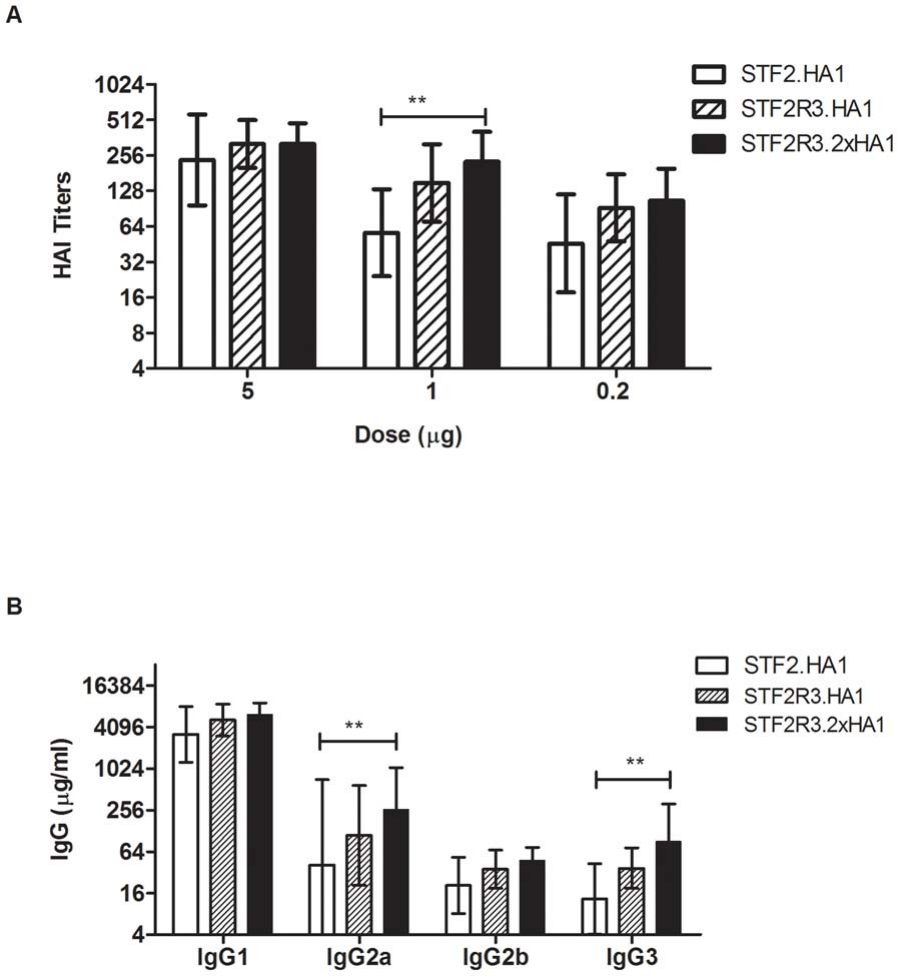
Antibody responses in BALB/c mice. Mice were immunized *s.c.* on days 0 and 21, and bled on day 42. Neutralizing antibody titers and HA-specific IgG subclasses were measured by HAI test (**A**, n = 10) and ELISA (**B**, (n = 5), respectively, and expressed as GMTs ±95%CI. **, *p*<0.01 in 2-way ANOVA/Bonferroni test.

In our initial immunogenicity experiment, groups of 10 BALB/c mice were immunized *s.c.* twice at a 3-week interval with doses of 5, 1 and 0.2 µg of STF2.HA1, STF2R3.HA1, and STF2R3.2xHA1. Post boost sera were prepared and subjected to an HAI test. The results in Fig. 3A indicated that all three formats elicited strong HAI responses. At the 1 µg dose level, STF2.HA1, STF2R3.HA1, and STF2R3.2xHA1 elicited HAI titers of ≥40 in 70% (GMT = 57), 90% (GMT = 149), and 100% (GMT = 226) of mice, respectively. At the sub-microgram (0.2 µg) dose, these candidates were also very immunogenic; STF2.HA1, STF2R3.HA1, and STF2R3.2xHA1 elicit a HAI titer of ≥40 in 60% (GMT = 46), 90% (GMT = 92), and 90% (GMT = 106) of mice, respectively. Overall, STF2R3.2xHA1 was significantly more effective than the STF2.HA1 format in eliciting neutralizing antibodies. In addition, the STF2R3.2x HA1 format is more active in inducing all subclasses of IgG, particularly IgG2a and IgG3 subclasses compared to the STF2.HA1 format (Fig. 3B).

**Figure 4:**
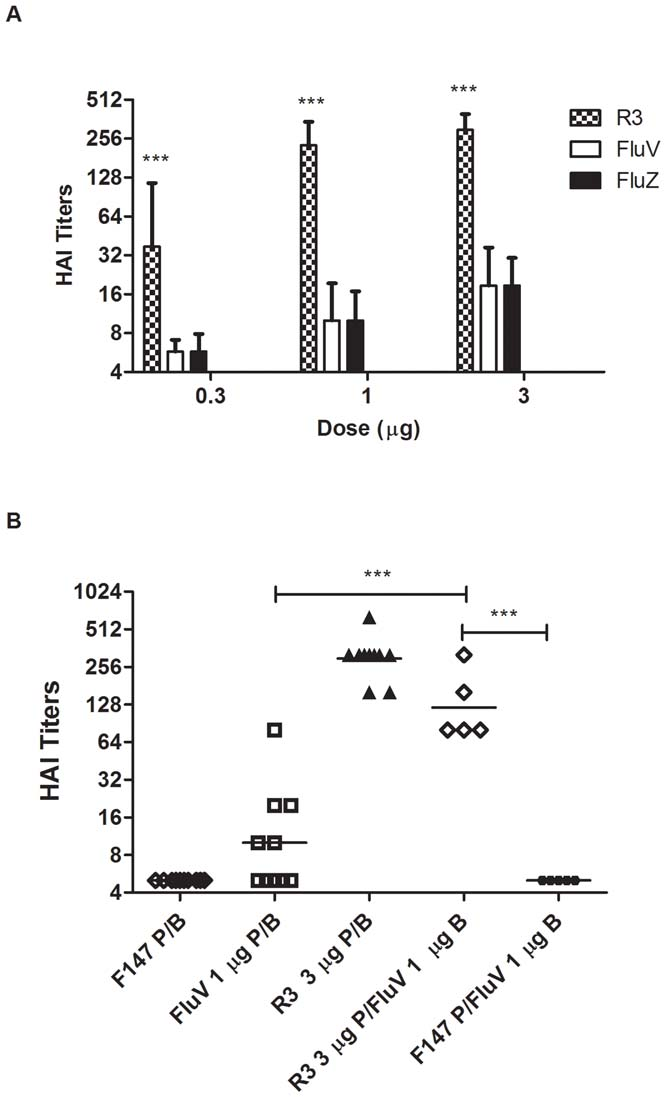
HAI titers of mice immunized with STF2R3.HA1, and/or TIV. Mice (n = 10) were primed (P) and boosted (B) with F147, STF2R3.HA1 (R3), Fluvirin (FluV), or Fluzone (FluZ) at the indicated doses on days 0 and 21, and bled on day 42 (**A**). Separate groups of mice (n = 5) were primed with 3 µg of STF2R3.HA1 or F147, and boosted with 3 µg Fluvirin (**B**). Neutralizing antibody titers were measured by HAI test, and expressed as GMTs +95%CI (A) or GMTs (B). Doses of Fluvirin and Fluzone are given according to the contents of H1N1 HA. ***, *p*<0.001 in 2-way ANOVA/Bonferroni test (**A**) or 1-way ANOVA/Tukey test (**B**).

To compare the immunogenicity of our vaccine with commercial TIVs, we selected the intermediate STF2R3.HA1 candidate, which can be easily purified at the highest yield among all three candidates. We immunized mice with Fluvirin (Novartis), Fluzone (Sanofi-Aventis), or STF2R3.HA1 at different doses. HAI titers in Fig. 4A demonstrate that STF2R3.HA1 elicits significantly higher HAI titers than Fluvirin or Fluzone. Specifically, the 1 µg dose of STF2R3.HA1 elicited a GMT titer of 226; whereas Fluvirin at 9 µg (containing 3 µg of H1 HA) induced a GMT titer of 19. The H1N1 component of another TIV (Fluzone, Sanofi-Aventis) displayed equally low immunogenicity, as 2 *s.c.* immunizations with Fluzone at 1, 3, and 9 µg (containing 0.3, 1, and 3 µg of H1 HA) led to geometric mean HAI titers of 6, 10, and 19, respectively (Fig. 4A). These results indicate that the STF2R3.HA1 format candidate vaccine is more immunogenic than the H1N1 component of two commercial TIVs. Furthermore, mice primed with STF2R3.HA1 and boosted with Fluvirin generated a significantly higher HAI titer (GMT = 121) than those primed and boosted with Fluvirin (GMT = 10) (Fig. 4B).

**Figure 5:**
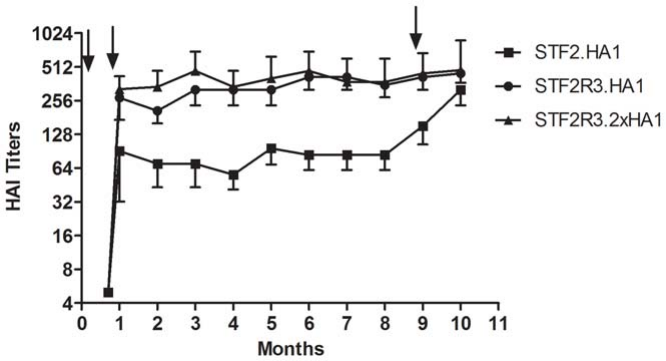
Longevity of neutralizing antibody response. Mice (n = 5) were immunized *s.c.* with the indicated candidates (1 µg/mouse), and bled monthly for 10 months. Serum neutralizing antibody titers were measured by HAI assay and expressed as GMTs +95%CI. Immunization time points (days 0, 21 and 266) are given in arrows.

We next examined the durability of the antibody responses induced by these vaccine candidates. No neutralizing antibody was detected in mice following priming with any of these candidates (Fig. 5). After one booster immunization, all three candidates, particularly the STF2R3.HA1 and STF2R3.2xHA1, elicited HAI antibody responses that lasted for at least 8 months after the boost. These mice, however, did not show a significant anamnestic antibody response measured as a rise in HAI titers. In contrast, mice responded to the first STF2.HA1 boost to a lesser extent, but showed a stronger memory response following the second booster immunization (Fig. 5).

**Figure 6:**
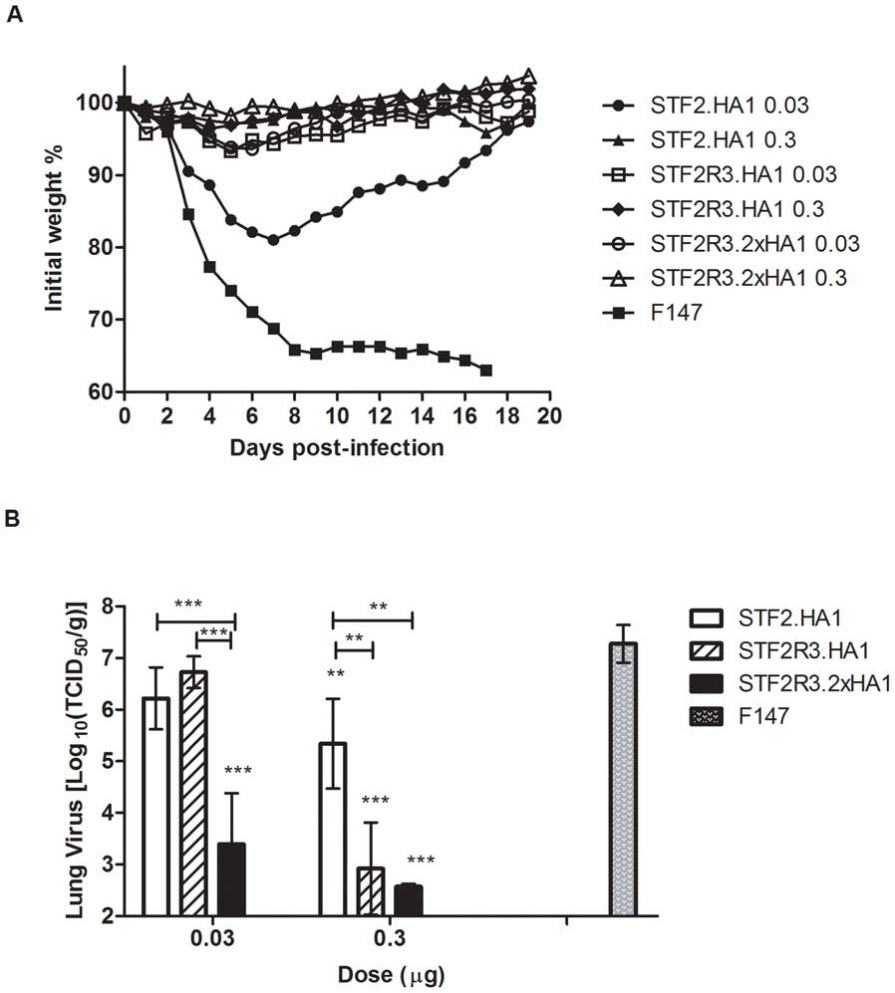
Effects of immunization on mean weight changes and day 4 lung virus titers of infected mice. Mice (n = 15) were immunized *s.c.* on days 0 and 21, and challenged I.N. with A/California/04/2009 on day 42 (experiment 2 in Table 1). Infected mice were monitored daily for weight change for 21 days. Four days post infection, lungs were collected from selected groups and subjected to TCID_50_ test. (**A**) Mean weight change (n = 10): a very significant difference was found in the mean weight change for all the vaccinated groups compared to the placebo (*p*<0.001) in 1-way ANOVA/Tukey test. (**B**) Lung virus titers (n = 5, means ± SDs) **, *p*<0.01, ***, *p*<0.001 in 2-way ANOVA/Bonferroni test.

Two mouse immunogenicity and efficacy experiments were conducted at Utah State University. Microgram and sub-microgram doses (prime and boost) of all three vaccine formats provided full protection against weight loss and death following a lethal viral (mouse-adapted A/California/04/2009) challenge (Fig. 6A and Table 2). Similar to earlier results, in these studies the immunogenicity of the candidates was ranked as STF2R3.2xHA1>STF2R3.HA1>STF2.HA1 (Table 2). Post viral challenge, mice (n = 10) were monitored for weight change and survival. Five additional mice from selected groups were euthanized four days post-infection to determine lung virus titers. At the discriminating 0.03 µg dose level, the survival rates of the STF2.HA1, STF2R3.HA1 and STF2R3.2xHA1 groups are 20–90%, 60–100%, and 90–100%, respectively. In the second efficacy experiment, mice receiving the STF2.HA1 lost a significantly higher percentage of weight compared to those receiving the STF2R3.HA1 and STF2R3.2xHA1 candidates (*p*<0.001, 0.03 µg/mouse) (Fig. 6A). The survival rates are associated with 1.1, 0.6, and 3.9 log10 unit reduction in lung virus titer for STF2.HA1, STF2R3.HA1, and STF2R3.2xHA1, respectively (Fig. 6B). A more dramatic reduction (4.3–4.7 log units) in lung virus titers was seen for the 0.3 µg dose groups receiving the STF2R3.HA1 and STF2R3.2xHA1 candidates. A two log unit reduction in lung titers was found in mice immunized with STF2.HA1 (0.3 µg). The weight loss and lung virus titer data showed a parallel pattern (Fig. 6A & 6B). Therefore, our data indicate that while all three formats are protective, the relative effectiveness of the three vaccine formats appears to be in the order of STF2R3.2xHA1>STF2R3.HA1>STF2.HA1.

**Table 2:** HAI titers and survival rates of immunized mice.

Vaccines	Dose (µg)	Experiment 1		Experiment 2	
		GMT (SP)	Survival (%)	GMT (SP)	Survival (%)
STF2.HA1	3	116 (93)	100***	91 (64)	100***
	0.3	17 (33)	100***	19^c^ (44)	100***
	0.03	5 (0)	20	6^d^ (0)	90**
STF2R3.HA1	3	139 (93)	100***	300 (100)	100***
	0.3	30^a^^,^ b (60)	100***	53 (67)	100***
	0.03	6 (0)	60*	10 (7)	100***
STF2R3.2xHA1	3	211 (93)	100***	234 (100)	100***
	0.3	88 (93)	100***	153 (87)	100***
	0.03	10 (20)	90*** ^,^ e	22 (33)	100***
F147	N/A	5 (0)	0	6 (0)	10

Mice were immunized *s.c.* on days 0 and 21, bled on day 35, and challenged I.N. with 500 TCID50 of mouse adapted A/California/04/2009 on day 42. Infected mice were monitored daily (n = 10) for mortality for 21 days. SP: seroprotective titer, mice% with ≥40 HAI titers.

Two-way ANOVA/Boferroni tests for HAI data (n = 15):

a , *p*<0.01 (**) vs STF2.HA1;

b , *p*<0.001 (***) vs STF2R3.2xHA1;

c , *p*<0.001 (***) vs STF2R3.2xHA1;

d , *p*<0.05 vs STF2R3.2xHA1.

Fisher's exact test for survival data (n = 10):

* , *p*<0.05;

** , *p*<0.01;

*** , *p*<0.001;

e , *p*<0.05 (*) vs STF2.HA1 at the same dose (0.03 µg/mouse).

## Discussion

VaxInnate has developed a flagellin fusion protein-based vaccine platform that effectively links innate and adaptive immunity. The flagellin portion of the fusion potentiates the immune response by triggering TLR5. The HA globular head contains the majority of HA neutralizing epitopes thereby eliciting a specific immune response against influenza virus. Induction of a potent antigen specific immune response requires the physical linkage of the antigen to flagellin, as co-delivery of the 2 components failed to induce highly protective immunity to influenza and flavivirus infections in mice [16], [17]. We have previously demonstrated the excellent immunogenicity and efficacy of this vaccine platform when applied to either seasonal H1N1 or highly pathogenic avian influenza H5N1 in animal models [14], [9]. More recently we have demonstrated that a seasonal H1N1 vaccine, VAX125, is well tolerated and immunogenic in man [15]. Importantly, this vaccine was extremely well tolerated and highly immunogenic in the typically poorly responsive elderly population (unpublished results). Here we report promising preclinical immunogenicity and efficacy results of three vaccine formats targeting 2009 pandemic H1N1 influenza. The antigenicity profile (STF2R3.2xHA1>STF2R3.HA1>STF2.HA1) of these three vaccine candidates is similar to that for previous STF2R3.HA5 and STF2.HA5 (H5N1) VN candidates [9]. These results confirm previous results and demonstrate that conformational epitopes of the HA globular head of these candidate vaccines are well preserved following the purification process.

All three vaccine candidates elicit robust and durable neutralizing antibody responses for at least 8 months in mice (Fig. 5). Following a second booster immunization at month 9, mice receiving the STF2.HA1 candidate displayed a better anamnestic antibody response. Higher pre-existing HAI titers in the STF2R3.HA1- and STF2R3.2xHA1- immunized mice as seen with TIV vaccinations in humans [18] may have contributed to the lower fold increases after the third immunization.

Infection with influenza virus or immunization with a live attenuated influenza vaccine in BALB/c mice elicits predominantly IgG2a or IgG1 responses [19]. VaxInnate's flagellin fusion vaccine candidates elicit all four subclasses of IgG with a Th2-type response characterized by high levels of IgG1 antibodies in BALB/c mice. This is expected for a subunit protein vaccine. In addition, the STF2R3.2xHA1 format elicits significantly higher levels of IgG2a and IgG3 compared to the STF2.HA1 format. While IgG1 has been suggested as the most important subclass of neutralizing antibodies, IgG2a antibodies have been shown to contribute equally to hemagglutination inhibition *in vitro* and virus elimination in BALB/c mice [19]. The higher levels of IgG2a are likely to correlate to an increased viral clearance due to its Fc receptor (e.g., ADCC) and complement activation [20], [21]. The higher levels of IgG3 observed in the STF2R3.2xHA1 groups could be associated with T cell independent antibody induction in mice.

We have demonstrated that attachment of the HA globular head at different positions of the flagellin differentially potentiates antibody responses, resulting in differences in immunogenicity and efficacy between vaccine candidates (STF2R3.2xHA1>STF2R3.HA1>STF2.HA1). The immunogenicity profile of these vaccine formats appears to correlate well to that of protective efficacy in mice. We find a similar immunogenicity profile in rabbits (unpublished results). Mice, following intranasal influenza inoculation, develop lethal pneumonia, and virus replicates to very high titers in the lungs of infected mice. Although other mechanisms such as IgA and cytolytic T lymphocytes may have contributed to the excellent efficacy in this study, serum IgG transudated to the lung is likely to play a major role in protecting mice against lung infection and death [22]. Using the well established A/PR/8/34 model, we have been able to fully protect mice against a lethal virus challenge following passive transfer of immune sera (unpublished results).

Our data from comparative immunogenicity studies in mice of currently licensed TIVs and the STF2R3.HA1 candidate indicate that the STF2R3.HA1 format candidate is more immunogenic than the H1N1 component (A/California/07/2009) of two commercially available TIVs (Fluvirin and Fluzone, 2010/2011). The STF2R3.HA1 format was also found to prime mice better than Fluvirin, demonstrating the superior immunogenicity of VaxInnate's CA07 vaccine candidate in our preclinical models. This is presumably due to the immunopotentiating properties of flagellin.

The potential manufacturing advantages of VaxInnate's vaccine platform were demonstrated in response to the recent 2009 pandemic influenza when the world was facing challenges in timely delivery of conventional vaccines. VaxInnate's pandemic vaccine platform has significant advantages over the traditional egg-based flu vaccine manufacturing technology due to its rapid speed and low cost. The pandemic vaccine candidates tested in this report proved to be easy to manufacture using standard biopharmaceutical manufacturing equipment under cGMP conditions, resulting in highly pure vaccines (≥95%). At commercial scale, it is anticipated that over 150 million doses of these vaccines could be manufactured under cGMP conditions from a single 600 L bioreactor batch, thereby allowing bulk delivery of the equivalent of 300 million doses of pandemic vaccine within 4–5 months. These vaccine candidates showed an excellent safety and immunogenicity profile in recent Phase I studies (manuscript in preparation). Recently, VaxInnate has applied the same flagellin fusion vaccine platform to several other potential pandemic influenza strains including H2N3, H5N1 (Indonesia), H7N2, H7N7, and H9N2 subtypes. Preliminary data from these studies demonstrated the general suitability of this vaccine platform as a very promising next generation influenza vaccine technology.

In conclusion, VaxInnate's pandemic H1N1 vaccine candidates elicit robust, long-lasting neutralizing antibodies in animal hosts and are highly effective in protecting mice against lethal viral challenge. Furthermore, attachment of the HA globular head at different positions of the flagellin differentially potentiates antibody responses, resulting in differences in immunogenicity and efficacy between vaccine candidates. These candidate vaccines are under clinical development.

## Materials and Methods

### Ethics statement

The mouse immunogenicity studies were conducted in accordance with the approval of the Institutional Animal Care and Use Committee (IACUC) of Princeton University dated 17 March 2010. Princeton University has held AAALAC accreditation since October 10, 2002. The U. S. Government (National Institutes of Health) assurance was renewed 31 March 2008 for a period of four years (PHS Assurance No. A3434-01) in accordance with the National Institutes of Health Guide for the Care and Use of Laboratory Animals.

The mouse efficacy studies were conducted in accordance with the approval of the IACUC of Utah State University dated 20 September 2010. The work was done in the AAALAC-accredited Laboratory Animal Research Center of Utah State University. The U. S. Government (National Institutes of Health) approval was renewed 1 April 2010 (PHS Assurance No. A3801-01) in accordance with the National Institutes of Health Guide for the Care and Use of Laboratory Animals (Revision; 2010).

### Cloning of recombinant vaccine candidates

Recombinant flagellin-HA globular head genes of the STF2.HA1 and STF2R3.HA1 candidates were designed and constructed as previously described [9]. Briefly, codon optimized synthetic genes of the hemagglutinin (HA) globular head of A/California/04/2009 fused to the C-terminus, R3 position, or both positions of the *Salmonella typhimurium* fljB (flagellin phase 2), STF2 gene were cloned into the pET24a vector to generate the constructs STF2.HA1 (CA04), STF2R3.HA1 (CA04), and STF2R3.2x.HA1 (CA04). Subsequently, STF2.HA1 (CA07) and STF2R3.HA1 (CA07) constructs corresponding to the A/California/07/2009 isolate were generated by mutating a single amino acid residue in the HA globular head of the corresponding CA04 plasmids.

The plasmid encoding STF2R3.2×HA1 (CA07) was made from a fusion of the DNA from the STF2R3.HA1 (CA07) and the STF2.HA1 (CA07) plasmids. For this, both plasmids were digested separately with *Nde*I and *Mfe*I enzymes. Specific fragments of the STF2R3.HA1 (CA07) (as insert) and STF2.HA1 (CA07) (as vector) were gel purified and ligated to form a complete DNA sequence encoding STF2R3.2xHA1 (CA07). The common restriction site is in the N terminal section of STF2. The DNA sequence of all plasmids was confirmed by Genewiz Inc. (South Plainfield, NJ). For protein expression, sequence confirmed plasmids were transformed into BL21 (DE3) strain of *E. coli* cells (Novagen, San Diego, CA). Cell lysates were evaluated by 4–12% NuPAGE (Invitrogen, Carlsbad, CA), individual protein bands corresponding to the expected molecular weights of the vaccine candidates were easily visualized by Coomassie Blue staining and Western blot analyses.

### Protein expression and purification

All three proteins (STF2.HA1, STF2R3.HA1, and STF2R3.2xHA1) were expressed and purified using similar process steps modified from those previously described [9]. Briefly, target protein was expressed in batch bioreactor cultures and isolated to high purity using standard biopharmaceutical processes. Bulk protein was formulated by dilution into formulation buffer for subsequent testing.

The fermentation processes were identical for the three proteins. Banked cells were cultured in shake flasks using a modified LB media and inoculated into a bioreactor containing similar media. After a period of growth controlled for pH, temperature and dissolved oxygen, cells were induced by IPTG and harvested.

Target protein was purified using a standard protocol with minor variations between the proteins. After harvest, protein was released from the cells by homogenization and purified by clarification, refolding/capture chromatography, polishing chromatography, and bulk formulation. STF2.HA1 is mostly expressed as a soluble protein and was clarified primarily by precipitation (Table 1). STF2R3.HA1 and STF2R3.2xHA1 are expressed as inclusion bodies and were clarified by inclusion body washing (Table 1). After clarification, proteins were resolubilized in urea buffer, refolded by rapid dilution, and captured by binding AEX chromatography (Super Q, Tosoh Biosciences LLC., King of Prussia, PA). Post-capture polishing purification was performed by size exclusion chromatography (Superdex 2000, GE Healthcare BioSciences, Piscataway, NJ) using F147 formulation buffer (10 mM Tris, 10 mM Histidine, 150 mM NaCl, 5% Trehalose, 0.02%, polysorbate 80, 0.1 mM EDTA, 0.5% Ethanol, pH 7.0) as mobile phase. Resulting protein preparations were at least 90% pure by reverse phase chromatography, contained ≤2% aggregates, and contained low concentrations of endotoxin, host cell proteins, and nucleic acids.

### Viruses and cells

Influenza A/California/07/2009 virus was obtained from the US Center for Disease Control and Prevention (Atlanta, GA), amplified in 10-day old SPF research eggs (Charles River Laboratories Inc., Storrs, CT), and titrated by hemagglutination test using Turkey red blood cells (Lampire Biologicals Lab, Pipersville, PA). Mouse-adapted influenza A/California/04/2009 received from Dr. Elena Govorkova (Department of Infectious Diseases, St. Jude Children's Research Hospital, Memphis, TN) was propagated and titrated in MDCK cells at Utah State University. The virus was adapted to replicate in the lungs of BALB/c mice by 9 sequential passages through mouse lungs [23]. Virus was plaque purified in MDCK cells in minimal essential medium (MEM/EBSS, HyClone Laboratories, Inc., Logan Utah). A working virus stock was prepared by growth in embryonated hens' eggs and then MDCK cells.

### ELISA for measurement of antigenicity

ELISA plates were coated with the following antigens: recombinant STF2.HA1, STF2R3.HA1, STF2R3.2xHA1, and baculovirus expressed HA0 CA04 (Protein Sciences, Meriden, CT). Triplicate wells were coated with serial dilutions starting at 320 nM and incubated overnight at 4°C. Plates were blocked with 300 µl/well of SuperBlock T20 buffer (Thermo Fisher Scientific, Rockford, IL) for 2 hours at room temperature. Plates were washed three times with wash buffer (PBS supplemented with 0.05% Tween-20), ferret post A/California/07/2009 infection serum (1∶400 dilution, CDC) was added, and plates were incubated for 1.5 hr at room temperature. Plates were washed three times with wash buffer, HRP-conjugated goat anti-ferret IgG (h+l) (Bethyl Laboratories, Inc., TX) was added and the plates were incubated at room temperature for 45 min. After adding TMB-ELISA substrate and then stop buffer, the mean OD_450_ of triplicate wells was determined and graphed.

### ELISA for measurement of IgG subclasses in serum

ELISA plates were coated with 0.2 µg/well HA0 CA04 (Proteins Sciences, Meriden, CT) or serially diluted mouse polyclonal IgG standard. After washing, the plates were blocked with Assay Diluent (BD Biosciences, San Diego, CA) for one hour. Serially diluted mouse immune serum samples were added; and plates were incubated for 1 hour at room temperature. After washing three times with wash buffer, HRP-conjugated goat anti-mouse IgG (Jackson ImmunoResearch Labs, West Grove, PA) was added and incubated at room temperature for 45 min. After adding TMB-ELISA substrate and then stop buffer, the mean OD_450_ of duplicates was determined and graphed. Quantification (specific IgG in µg/ml) is performed using a standard curve of polyclonal IgG coated on the same plate, fitted with a 4-parameter logistic equation (Softmax 5.2, Molecular Devices, CA). Data are reported as results of individual mice.

### Hemagglutination inhibition (HAI) test

The HAI test was performed as previously described [14]. Post-boost serum samples were treated with receptor destroying enzyme (RDE) II overnight, heat-inactivated (56°C 45 min), diluted in 96-well V-bottom microtiter plates, and incubated with 4 HA units (HAU) of influenza A/California/07/2009 virus in 25 µl for 30 minutes at room temperature. Turkey red blood cells (TRBC, 0.5%) (Lampire Biological Laboratories, Pipersville, PA) were added (50 µl/well), mixed briefly, and incubated for 30–60 min at room temperature. The HAI titers of serum samples are reported as the reciprocal of the highest dilution at which hemagglutination was completely inhibited. Sheep anti-A/California/07/2009 serum (CBER, FDA) or ferret post infection anti-A/California/07/2009 serum (CDC) was used as reference serum to monitor the variability among different tests.

### TCID_50_ test

Mouse lungs were homogenized in 1 ml of MEM/EBSS medium and assayed for infectious virus in MDCK cells, as described previously [24]. Samples were serially diluted in 10-fold increments in 96-well plates of MDCK cells. Four wells were used per virus dilution. Cytopathic effects induced by the virus were determined at 3 and 6 days, with virus titers calculated by the method of Reed and Muench (1938) [25]. Fifty percent cell culture infectious doses (TCID_50_) were converted to TCID_50_ per gram of lung tissue prior to statistical analysis. The limit of detection for the TCID_50_ assay was 0.75 log_10_ unit.

### Immunogenicity studies

Immunogenicity studies were conducted at Princeton University following an IACUC-approved protocol. Groups of ten female BALB/c mice (6–8 week old, Charles River Lab, Wilmington, MA) were immunized subcutaneously (*s.c.*) on days 0 and 21 with 0.2–5 µg of STF2.HA1, STF2R3.HA1, and STF2R3.2xHA1 candidate vaccines, a commercial TIV Fluvirin (Novatis) or Fluzone (Sanofi-Aventis). Blood was collected via retro-orbital puncture on day 35 or 42. And serum samples were prepared following centrifugation, and stored at −20°C until use.

### Mouse efficacy studies

Mouse efficacy studies were conducted at Utah State University. Groups of 15 female BALB/c mice (6 week old) were immunized *s.c.* on days 0 and 21, and bled on day 35 to prepare immune sera. Animals were anesthetized with a ketamine (100 mg/kg)/xylazine (5 mg/kg) mixture and intranasally challenged with mouse-adapted A/California/04/2009 (500 TCID_50_ in a 90-µl volume per mouse) on day 42. Animals were monitored daily for morbidity and mortality for 21 days. Four days after the viral infection, five mice from selected groups were euthanized; lung samples were collected to measure the virus titers by TCID_50_ assay.

### Statistical analyses

The titers of neutralizing antibodies and virus titers were log10-transformed, and subjected to 1-way ANOVA or 2-way ANOVA analysis with Tukey or Bonferroni post tests using GraphPad Prism 5.00 (GraphPad Software, San Diego, CA). Survival curves between different groups were compared with Fisher's exact test. The mean body weights were analyzed by ANOVA followed by Tukey's multiple comparison test using Prism 5.0.
